# Determination of insensible water loss and sodium accumulation behavior from the Medtronic Nautilus Extracorporeal Membrane Oxygenation (ECMO) oxygenator: An in vitro study

**DOI:** 10.1051/ject/2025026

**Published:** 2025-12-17

**Authors:** Carrie Whittaker Striker, Gabriel Kong

**Affiliations:** 1 Department of Cardiovascular Perfusion, Children’s Wisconsin Milwaukee WI USA; 2 Electrical, Computer and Biomedical Engineering Department, Milwaukee School of Engineering Milwaukee WI USA

**Keywords:** Extracorporeal membrane oxygenation, ECMO, Oxygenator, Insensible water loss, Fluid balance, Sodium, Hypernatremia

## Abstract

*Introduction*: Fluid and electrolyte balance is closely managed in extracorporeal membrane oxygenation (ECMO) patients. Neglecting oxygenator-related insensible fluid losses can distort fluid balance and electrolyte levels. While ECMO oxygenator insensible losses are reported, they remain undefined for the Medtronic Nautilus oxygenator. Through in vitro analysis of the Nautilus, we quantified insensible water losses while concurrently observing sodium behavior. *Methods*: Insensible water losses were determined using a closed circuit. A 12-hour pilot run was conducted to saturate the oxygenator and determine probable water loss rates and sodium accumulation behaviors. Fluid loss and sodium measurements were made at 0, 6, and 12 h. Immediately following the pilot run, three randomly assigned sweep gas rates, 0.5, 1.0, and 1.5 L/min, were evaluated over a 24 h period and replicated in triplicate. The circuit parameters were consistent and controlled for each trial. Data were collected at 0, 12, and 24 h for visualized water loss in the reservoir. After each trial, sterile water was introduced into the circuit via syringe and recorded as replacement volume. Sodium measurements were made for three trials (0.5, 1.0, and 1.5) and collected at 0 and 24 h. *Results*: Using linear regression analysis, the following insensible water loss equations were produced. Visualized volume: 2.74 mL/h per 1 L/min sweep gas rate or 65.66 mL/day per 1 L/min of sweep gas rate (*p* < 0.001). Replacement volume: 3.02 mL/h per 1 L/min of sweep gas rate or 72.5 mL/day per 1 L/min of sweep rate (*p* < 0.001). Sodium accumulation was observed, but not statistically significant due to the small sample size. *Conclusion*: Insensible water loss in the Nautilus ECMO oxygenator increases linearly with sweep gas rate (*p* < 0.001), leading to sodium accumulation through evaporation. These losses and the associated risk for hypernatremia should be considered when managing a patient’s fluid and electrolyte balance on ECMO.

## Introduction

Extracorporeal Membrane Oxygenation (ECMO) is a life-supportive therapy utilized for critically ill patients who do not adequately respond to conventional cardiac and/or respiratory medical interventions. Given the severity of illness in this population, meticulous attention to fluid and electrolyte balance is crucial [1]. Neonatal and infant patients are particularly susceptible to major fluid shifts upon commencing ECMO due to their diminutive circulating volume compared to the ECMO circuit volume [2]. Fluid overload for patients on ECMO has been associated with poorer outcomes such as increased mortality, duration of mechanical ventilation, and intensive care unit (ICU) length of stay [3, 4]. Furthermore, increases in serum sodium can be observed due to sodium-based volume replacement fluids in the ICU and evaporative losses, such as the insensible water losses from ventilation of the ECMO oxygenator [2, 5, 6]. Since oxygenator evaporative losses are directly related to sweep gas rate, this observation is compounded for ECMO patients experiencing hypercarbia, where higher sweep rates are necessary [1, 2, 7–9]. In pediatric ICU patients, sequelae of hypernatremia include hyperosmolality, neurologic complications, acute kidney injury, and increased length of ICU stay and mortality [10–12].

A patient’s fluid balance is typically derived utilizing easily measured inputs and outputs; however, difficult-to-measure losses, such as evaporative or insensible water losses from the ECMO oxygenator, also contribute to the patient’s fluid status [4]. Excluding these losses may result in a falsely positive fluid balance, leading to interventions that may be unnecessary or even detrimental to the patient [4]. Examples might include administration of diuretics or utilization of therapies to remove excess fluid, like continuous renal replacement therapy (CRRT), slow continuous ultrafiltration (SCUF), and/or continuous veno-veno hemofiltration (CVVH) [4]. Concomitantly, unnecessarily lowering the patient’s circulating volume may drop ECMO flow rates below clinically desired thresholds due to the preload dependency of the circuit [13].

In vivo and in vitro studies evaluating evaporative losses with ECMO oxygenators have been established in the literature [1, 2, 4, 7–9, 14]. These studies consist of ECMO oxygenators of various construction and materials, marking the changes in materials over time, including silicone, micro-porous hollow fiber, and polymethylpentene (PMP) membranes. Each of these membrane types exhibits insensible water losses that linearly correlate to the rate of ventilatory gas, or sweep gas, passing through the oxygenator [1, 2, 4, 7, 8]. Li Li et al. determined that the fluid temperature, in addition to sweep gas rate, is linearly correlated to insensible water losses [9]. Alexander et al. demonstrated that circuit sodium concentrations increased with higher sweep rates and resultant insensible water losses with a microporous hollow fiber oxygenator [7]. Due to the clinical necessity of evaluating a comprehensive fluid and electrolyte balance for patients undergoing ECMO, it is necessary to evaluate the most current oxygenator devices for insensible water loss and sodium accumulation behaviors.

The Medtronic Nautilus ECMO oxygenator (Minneapolis, MN, USA) is intended for long-term use up to 48 h and has a PMP membrane with a surface area of 1.8 m^2^ [15]. The static prime is 226 mL and can support blood flow rates of 0.5–7 L/min. The sweep gas to blood flow ratio is 0.5:1 to 3:1 L/min [15].

Previous studies demonstrate varying results of insensible water losses with PMP membranes. Lawson and Holt measured 48 mL/day per L/min of sweep gas with the Jostra Quadrox D (Maquet, Bridgewater, NJ, USA) PMP device [8]. When evaluating the Hilite 2400LT PMP membrane (Medos, Medizintechnik AG, Stolberg, Germany), Gill and O’Shaughnessy demonstrated daily losses of 72 mL/day per L/min sweep gas [2]. The results from the Hilite device are more in line with previous studies, which evaluated insensible water losses in silicone membranes (72 mL/day per L/min sweep gas) and microporous hollow fiber membranes (82.7 mL/day per L/min sweep gas) [1, 7]. The aim of the study is to describe the insensible water loss rate and sodium accumulation behavior with the Nautilus oxygenator, at blood flows and sweep gas rates that mimic the neonatal/infant patient, over a 24 h.

## Materials and methods

### Circuit setup

**Figure 1:**
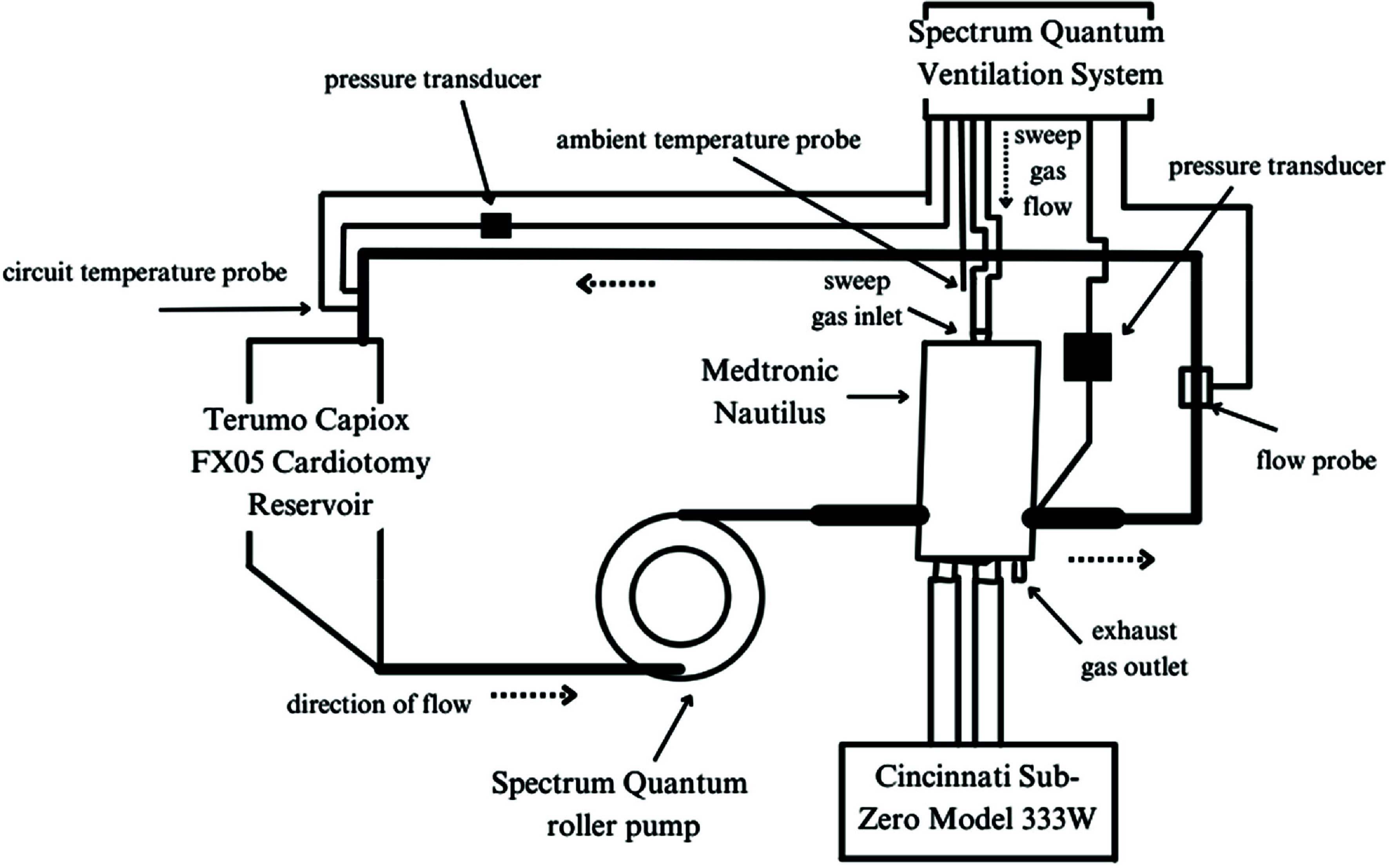
Schematic of the in vitro experimental setup.

An in vitro analysis was conducted using a Spectrum Heart-Lung machine (Spectrum Medical, Fort Mill, SC, USA) with an extracorporeal circuit consisting of a Medtronic Nautilus oxygenator with Balance biosurface and an integrated heat exchanger. To monitor volume changes in the circuit, a Terumo Capiox FX05 Reservoir with integrated cardiotomy and Xcoating (Terumo Cardiovascular, Ann Arbor, MI, USA) was used (Fig. 1). Both the Medtronic Balance biosurface and Terumo Xcoating are non-heparin polymer coatings designed to reduce platelet adhesion and protein denaturation [15, 16]. A Spectrum Quantum roller pump with Terumo 1/4″ ID × 3/32″ wall polyvinyl chloride (PVC) tubing was used to generate fluid flow. Between each trial, the roller pump tubing was marked with a permanent marker and shifted to distribute wear along the length of the tubing. After visible wear was observed on the exterior of the roller tubing, it was replaced with identical product and specifications. Due to the size difference between the oxygenator inlet and outlet ports (3/8″) and the reservoir inlet and outlet ports (1/4″), 1/4″–3/8″ Medtronic connectors were used to connect lengths of 1/4″ ID × 3/32″ wall and 3/8″ ID × 3/32″ wall Terumo PVC tubing with Xcoating.

**Figure 2:**
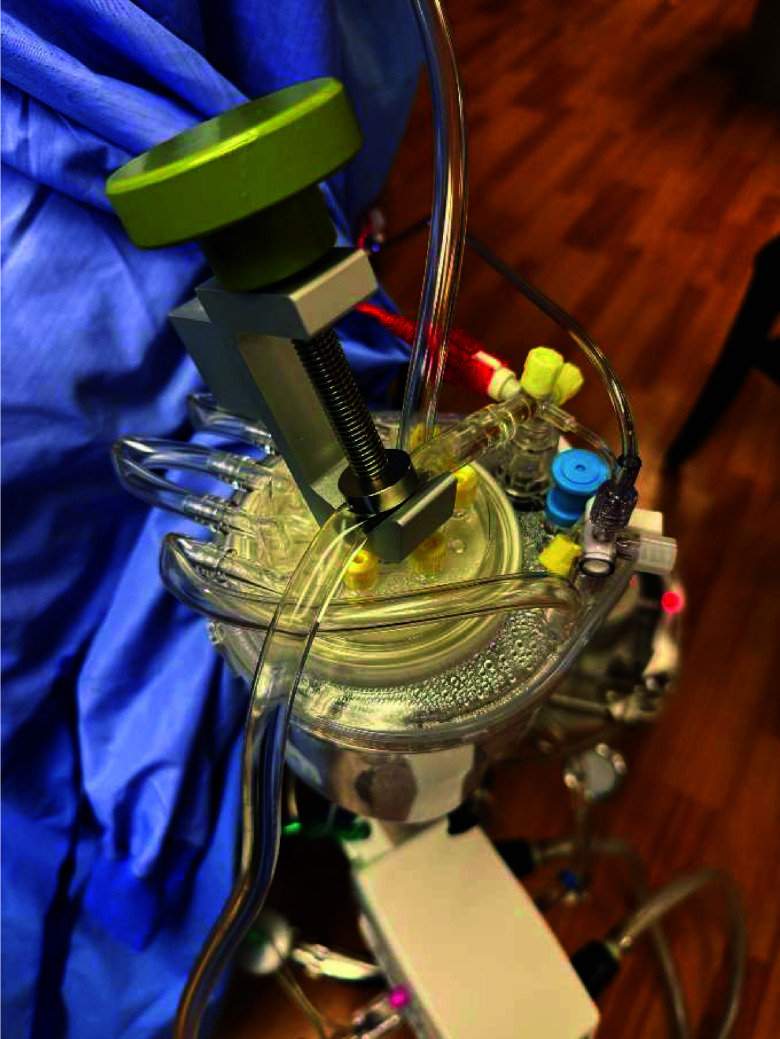
Reservoir with closed ports to prevent evaporative losses from the circuit and a partial occlusion clamp for generating afterload.

A gross occlusion was set for the roller pump and initially primed with 0.9% sodium chloride (Baxter Healthcare, Deerfield, IL, USA). The circuit flow rate was measured by a Spectrum H7XLH ultrasonic flow probe. Post-oxygenator pressure was generated with the screw-down clamp adapted from a Terumo Capiox sampling manifold holder by partially occluding the 1/4″ portion of the circuit post oxygenator (Fig. 2). Afterload pressure was measured via a luer port on the Nautilus outlet with a steady-state TruWave transducer (Edwards Lifesciences, Irvine, CA, USA) and was set at an average of 150 mmHg for all trials. Circuit temperatures were maintained at 36.5 °C with the Nautilus integrated heat exchanger and a Cincinnati Sub-Zero ECMO Heater Model 333 W (Cincinnati Sub-Zero Products, Cincinnati, OH, USA) with a set temperature of 37.3 °C. The temperature of the fluid was measured at the venous inlet of the reservoir utilizing a Terumo Capiox thermistor probe. Ambient temperature was measured near the reservoir with a Sorin non-sterile temperature probe (Sorin Group USA, Arvada, CO, USA).

Pressure, flow, and temperature were displayed on the Spectrum Quantum Workstation. Sweep gas was delivered to the oxygenator using the Spectrum Quantum Ventilation System and was set at an F_i_O_2_ of 21% for all trials, with varying sweep rates consistent with the trial.

The inlet ports on the reservoir were connected end-to-end with lengths of Terumo 3/16″ PVC tubing, and all luer ports were capped to prevent evaporative losses to the environment (Fig. 2). The static prime of the circuit was 325 mL with a reservoir starting level of 0 mL. Following each trial, sterile water was used to restore the reservoir volume to the starting level and to rebalance the circuit’s sodium concentration. All trials utilized the same oxygenator, and the experiment was conducted in a temperature-controlled environment.

### Pilot study

A pilot study was conducted to assess the general relationship between fluid loss and gas sweep rate over time and to thoroughly saturate or “wet out” the Nautilus oxygenator. A sweep gas rate of 5 L/min was utilized, and the fluid flow rate, afterload pressure, and circuit temperature were maintained as described in the circuit setup. A starting reservoir volume of 300 mL was used based on Lawson and Holt’s insensible water loss findings in their evaluation of the adult Quadrox D PMP ECMO oxygenator [8]. They suggested an insensible water loss rate of 48 mL/day per 1 L/min of sweep rate [8]. Applying that assumption, a fluid loss of 240 mL for a sweep gas rate of 5 L/min would be expected; however, the loss rate experienced over 12 h was higher, which resulted in early termination of the pilot study. Fluid losses were visually examined and recorded at 0, 6, and 12 h. At the end of the experiment and after recording the fluid level, the investigator drew a 50 mL waste and a 0.5 mL sample via a three-way stopcock from the Nautilus inlet port. The 50 mL waste was drawn to ensure that the sample accurately reflected the sodium level of the circuit and was analyzed by the ABL90 FLEX PLUS (Radiometer America, Brea, CA, USA). The waste fluid was reintroduced to the circuit after the sample was analyzed. Volume loss was also evaluated by the replacement method, where fluid was administered to the circuit utilizing a syringe until the volume in the circuit reached the starting reservoir level. Both methods of fluid loss were recorded.

### Study procedure

This study evaluated three sweep rates (0.5, 1.0, and 1.5 L/min) over 24 h. Each sweep rate was evaluated in triplicate for a total of nine 24-hour trials. Using a random number generator, the study order was determined for the nine trials (RANDOM.ORG, Dublin, Ireland). The circuit flow rate was set at 500 mL/min for all trials, and the circuit parameters were maintained as described in the circuit setup. The circuit flow and sweep gas rates were selected to mirror clinical conditions for neonatal patients.

Based on the pilot study results, a reservoir starting level of 200 mL was used. Fluid losses were visually observed by assessing the reservoir level at 0, 12, and 24 h. After 24 h, the sodium concentration of the circuit was analyzed. A 50 mL waste and a 0.5 mL sample were extracted from the circuit via a three-way stopcock from the oxygenator inlet port. The sodium concentration was measured using an ABL90 FLEX PLUS. Sodium values were assessed at 0 and 24 h. The waste fluid was reintroduced to the circuit after the sample was analyzed. Due to supply limitations, sodium accumulation data were evaluated for only three trials (0.5, 1.0, and 1.5 L/min), of which the initial and final sodium values were obtained. Additionally, following each trial, sterile water was introduced into the circuit using a syringe to achieve the trial starting level of 200 mL. This volume was recorded as a replacement volume.

### Statistical analysis

Computerized statistical analysis was conducted using Minitab version 22.2.1 (Minitab, LLC, State College, PA, USA) for Windows (Microsoft Corporation, Redmond, WA, USA). Kruskal-Wallis tests were used to test the null hypothesis that median fluid loss rates were equal for all three sweep gas rates within the visualized and replacement volume data. A simple linear regression model was built with the replacement volume data to describe the daily water loss rate. A separate multiple linear regression model was built with the visualized reservoir volume data. Sweep gas rate, time, and the interaction between sweep gas rate and time were included in the multiple linear regression model. Measured data are presented as mean ± 1 standard error of the mean, and regression parameters as mean estimate ± standard error of the regression coefficient. The sodium data was not statistically evaluated due to the small sample size.

## Results

### Pilot study

During the pilot study, the expected insensible water loss was 240 mL/day at a sweep gas rate of 5 L/min. This estimation of insensible water loss was derived from Lawson and Holt’s insensible water loss equation from an in vitro study of the Quadrox adult oxygenator [8]. At 12 h, the insensible water loss observed was 182 mL. Given the starting level of 300 mL, the pilot study was discontinued due to insufficient reservoir volume. Table 1 demonstrates the visualized insensible water losses over time for the pilot study. An insensible water loss rate of 3.03 mL/h per 1 L/min of sweep gas was observed.

**Table 1:** Visualized insensible water loss and sodium accumulation of the pilot study at a sweep gas rate of 5.0 L/min.

Time (hours)	Heater/cooler temperature ( °C)	Cardiotomy reservoir temperature ( °C)	Reservoir level (mL)	Calculated insensible water loss (mL)	Sodium ion concentration (mmol/L)
0	36.7	34.5	300	0	156
6	37.5	36.5	200	100	N/A
12	37.4	36.2	118	182	>170

### Sweep gas trials

During the sweep gas trials, the average ambient room temperature was 23.02 ± 0.08 °C, the average venous reservoir temperature was 36.39 ± 0.03 °C, and the average heater cooler temperature was 37.39 ± 0.03 °C. Both visualized and replacement volume data were collected to verify insensible water losses in the circuit.

### Visual assessment of insensible water loss

**Figure 3:**
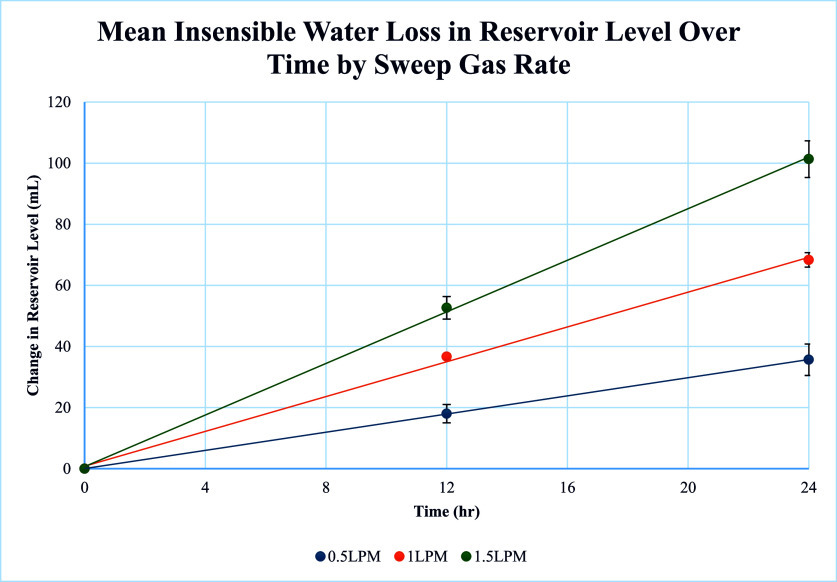
Mean insensible water loss observed over 24-hours per sweep gas rate.

Using a visual assessment of the reservoir level to assess fluid loss, a linear relationship was observed between fluid loss and time for each sweep gas rate tested (Fig. 3). A positive correlation was observed between fluid loss and sweep gas rate (*r* = 0.744). The multiple linear regression equation (Eq. (1)) retained the interaction between sweep gas rate and time (coefficient 2.736 ± 0.241, *p* < 0.001) and had an *R* ^2^ of 98.08%.

(1) $$\mathrm{Hourly}\;\mathrm{insensible}\;\mathrm{water}\;\mathrm{loss}\;\left(\;\mathrm{mL}\right)=2.736\times \mathrm{Sweep}\;\mathrm{gas}\;\mathrm{rate}\;\left(L/\min\right)\times \mathrm{Time}\;\left(h\right)$$

Using the coefficient from equation (1), an equation can be written to estimate the daily fluid loss based on the visualized results (Eq. (2)).

(2) $$\mathrm{Daily}\;\mathrm{fluid}\;\mathrm{loss}\;\left(\mathrm{mL}\right)=65.66\times \mathrm{Sweep}\;\mathrm{gas}\;\mathrm{rate}\;(L/\min)$$

The visual analysis of the reservoir level resulted in a mean total fluid loss over 24 h of 35.67 ± 5.17 mL at 0.5 L/min sweep gas, 68.33 ± 2.40 mL at 1.0 L/min sweep gas, and 101.33 ± 6.01 mL at 1.5 L/min sweep gas.

### Replacement volume assessment of insensible water loss

Using the replaced volume to assess fluid loss, a positive correlation (*r* = 0.989) was observed between sweep gas rate and fluid loss. The simple linear regression equation (Eq. (3)) only retained the sweep gas rate (coefficient 72.50 ± 4.11, *p* < 0.001) and had an *R* ^2^ of 97.80%. This equation can be used to estimate fluid loss over 24 h at a specified sweep gas rate.

(3) $$\mathrm{Daily}\;\mathrm{insensible}\;\mathrm{loss}\;\left(\mathrm{mL}\right)=72.50\times \mathrm{weep}\;\mathrm{gas}\;\mathrm{rate}\;\left(L/\min\right)$$

With the replaced volume method, the mean total fluid loss over 24 h was 34.67 ± 4.18 mL at 0.5 L/min sweep gas, 69.00 ± 1.15 mL at 1.0 L/min sweep gas, and 107.17 ± 5.35 mL at 1.5 L/min sweep gas.

### Sodium accumulation behavior

Sodium concentrations were evaluated at 0 and 24 h for three sweep gas trials (0.5, 1.0, and 1.5 L/min). For the 1.0 L/min trial, the final sodium concentration after 24 h exceeded 170 mmol/L, the upper limit of the ABL90. Sodium accumulations were observed, but establishing statistical significance was not possible due to the small sample size. Sodium accumulation data are presented in Table 2.

**Table 2:** Initial and final circuit sodium ion concentration levels for each sweep gas rate. Data are presented as single values.

Sweep gas rate (L/min)	Circuit sodium ion concentration (mmol/L)	
	Initial	Final
0.5	152	170
1.0	147	>170*
1.5	118	135

* The final value for the 1.0 L/min trial exceeded the upper limit of the ABL90 FLEX PLUS.

## Discussion

Fluid overload, or a positive fluid balance, is a common complication of ECMO [13, 17]. Neonatal and pediatric ECMO patients with fluid overload experience multiple adverse outcomes, including increased ECMO duration and mortality [17]. Managing an accurate fluid balance, including sources of insensible losses, is essential for properly assessing a patient’s fluid status and determining the most appropriate treatment approach. Recognizing the contribution of insensible water loss from the ECMO oxygenator may influence clinical management and potentially affect patient outcomes if left unaccounted for.

Evaporative losses experienced with oxygenators have been mentioned in the literature starting in 1996, where Visser and de Jong described evaporative losses with hollow fiber oxygenators [14]. Camacho et al. evaluated silicone membrane oxygenators and reported that insensible water loss was not related to fluid flow rate or membrane size [1]. Li Li et al. reported that insensible water loss linearly correlates with fluid temperature, and patients maintained at a lower temperature will experience less water loss [9]. Common to all investigators is a dependent relationship between sweep gas rate and insensible water loss [1, 2, 7–9]. In summary, the rate of insensible water loss is directly related to the rate of sweep gas and temperature of the fluid passing through the oxygenator [1, 2, 4, 7–9]. Table 3 presents the findings and design parameters for these in vitro experiments.

**Table 3:** Summary of findings for previous in vitro studies on insensible water loss.

Study authors	Oxygenator/surface area membrane type	Fluid flow rate mL/min	Findings
	Sweep gas rate	Fluid loss per day per L/min sweep	
Camacho et al. [1]	Avecor / 0.4 and 0.8 m^2^ Silicone Membrane (Avecor Cardiovascular, Inc., Plymouth, MN, USA)	200, 400 mL/min	Membrane size and fluid flow rate did not affect water loss.
			Water loss increased significantly with increased sweep gas rate.
	0.5, 1.0, 2.0 L/min	72 ± 11.6 mL	
Alexander et al. [7]	Medtronic Minimax / 0.8 m^2^ Microporous hollow fiber (Medtronic Inc., Minneapolis, MN, USA)	300 mL/min	Ambient air temperature and relative humidity had no statistically significant effect on water loss.
			Linear relationship between time, water loss, and sodium accumulation.
	2, 5, 10 L/min	82.7 ± 2.2 mL	
Lawson and Holt [8]	Jostra Quadrox D / 1.8 m^2^ PMP (Maquet, Bridgewater, NJ, USA)	500 mL/min	The relationship between water loss and sweep gas rate is approximately linear.
	2. 5, 10 L/min	48.0 ± 2.1 mL	
Gill and O’Shaughnessy [2]	Hilite 2400LT / 0.65 m^2^ PMP (Medos, Medizintechnik AG, Stolberg, Germany)	1000 mL/min	Heating and humidifying sweep gas resulted in a significant reduction in insensible water loss compared to heat-only trials.
			No significant difference in average water loss (mL/hour) per L/min of gas flow between all sweep gas rate groups.
	1, 3, 4.8 L/min	72.4 ± 3.9 mL	No measurable amounts of electrolytes were detected in collected water loss from gas exhaust.
			Water loss linear and directly proportional to sweep rate.
Li Li et al. [9]	Jostra Quadrox D / 1.8 m^2^ PMP	500 mL/min	Water loss linearly correlated with sweep.
			Water loss linearly correlated with temperature from 33–39 °C.
	3, 5, 7 L/min	66.2 mL @ 37 °C	

This study was designed to evaluate the Medtronic Nautilus oxygenator under clinical conditions typical of neonatal ECMO. The circuit flow rate of 500 mL/min is the minimum listed blood flow rate for the device [15]. Although neonatal ECMO can require less flow, a bridge between the oxygenator and the venous line is often used to achieve the minimum flow rate for the device. The sweep gas rates utilized for this study were determined based on the recommended sweep gas rate to fluid flow rate ratios outlined in the Nautilus Instructions for Use [15]. The minimum recommended sweep gas rate to flow rate ratio is 0.5:1 and the maximum ratio is 3:1 [15]. The sweep gas rates evaluated were 0.5, 1.0, and 1.5 L/min.

A pilot study was conducted to evaluate the relationship between fluid loss and sweep gas rate and to saturate the oxygenator. The pilot study, intended to last 24 h, was terminated at 12 h due to higher-than-expected insensible water losses. Based on the observation by Suttles et al., that peak saturation of the adult Quadrox-ID PMP membrane was achieved at 10 h, the investigators concluded that the oxygenator was adequately saturated to immediately commence the trial phase of the study following the termination of the pilot study [4].

All fluid losses were assumed to result from evaporated water that diffused across the oxygenator membrane. Visser and de Jong state that variations in membrane material and structure can lead to differing fluid losses across membranes from various manufacturers [14]. They investigated a microporous polypropylene hollow fiber membrane, whereas this study focused on a PMP membrane. The manufacturing process of PMP membranes results in a microporous structure on the gaseous side and a hydrophobic surface layer on the blood side [18]. Since the immediate fluid-side layer has no obvious micropores, water transmission through a PMP membrane would occur through activated diffusion [18, 19]. In this process, water vapor dissolves into the fluid-side layer, migrates through the microporous structure along its concentration gradient, then evaporates from the gaseous side of the membrane [19].

As noted by Visser and de Jong, fluid loss from a closed system generates negative pressure within the system [14]. This was also observed in the transduced reservoir pressures, which ranged from −45 to +1 mmHg, and in the post-oxygenator pressures, which decreased by 10–30 mmHg between observations. To maintain the operational afterload pressure of ~150 mmHg, the clamp on the venous line was tightened after each observation.

### Study limitations

This study may have been limited by the subjectivity of data collection. Despite choosing a reservoir with smaller graduations and having the same investigator make data collection, there was the potential for error and variability when assessing the volume in the reservoir. This reservoir has 10 mL graduations below 100 mL, 20 mL graduations between 100–200 mL, and 50 mL graduations between 200–300 mL. Hence, visualizing small changes between graduations was subjective. The method utilized in this study mirrored that of previous investigators; however, insensible water losses were determined in other ways. Gill and O’Shaughnessy used a hanging scale to weigh the losses from fluid-filled burettes into their closed system via a weight-to-volume conversion of 1 g to 1 mL [2]. Li Li et al. used a camera positioned directly at a 50 mL syringe connected to their closed system to record losses from the syringe every 500 s over 100 min [9]. Although the methods in this study might be more subjective, the results are like those of these researchers [1, 2].

Additionally, the small sample size of the daily fluid loss data for both the visualized and replaced volume groups (*n* = 3) limited the accuracy of the Kruskal-Wallis test, so these results were not presented. The regression models, however, have significant coefficients and characterize insensible water losses similar to previous researchers who utilized the volume replacement method [1, 2, 7, 9].

Lastly, the use of a crystalloid prime versus a blood prime may have impacted the results of this study. It is unclear how blood may change the insensible water loss rate, but comparing the results of this study with an in vivo analysis by Suttles et al. suggests that it is similar [4]. Studies to verify that finding would be necessary.

## Conclusion

This study demonstrates that the Nautilus ECMO oxygenator experiences a daily loss rate of 72.5 mL per 1 L/min of sweep gas rate (*p* < 0.001). Additionally, with the insensible water loss, sodium accumulations were observed in the circuit fluid. Gill and O’Shaughnessy noted that the condensation from the oxygenator outlet did not contain sodium, but the literature has yet to confirm that sodium does not cross the PMP membrane with evaporative losses by evaluating the circuit fluid/composition [2]. Though not statistically powered to do so, this study illustrates that sodium accumulates in the circuit with evaporative loss and can result in hypernatremic conditions in an in vitro setting. Hypernatremia in ECMO and pediatric cardiac surgical patients is associated with poorer outcomes such as neurologic sequelae, acute kidney injury, prolonged ventilation, and mortality [10–12].

Additionally, the impact of water loss and sodium accumulation may be more significant in pediatric patients with hypercarbia, as higher sweep rates are required for carbon dioxide removal. Given the direct proportionality between insensible water loss and sweep gas rate, the insensible water loss per kilogram may be more pronounced in this population, which could increase the risk of hypernatremia. Attention to serum sodium levels, fluid replacement types, and falsely positive fluid balances is imperative. Concern for overtreatment of a falsely positive fluid balance may impinge on clinical improvement.

This study, along with others, remains consistent on the amount of water loss that is experienced with PMP membranes, with the exclusion of Lawson and Holt, who studied the Quadrox D and reported a daily insensible water loss of 48 mL per 1 L/min of sweep gas rate [8]. In contrast to Lawson and Holt’s findings, Gill and O’Shaughnessy ran a pilot study to compare the Quadrox D with the Hilite 2400LT oxygenator and found that the water loss was almost identical at 72.7 *versus* 72.5 mL/day per L/min gas flow [2]. In Suttles et al. in vivo analysis of insensible water loss during ECMO, utilizing both the adult and pediatric Quadrox ID, found that the daily insensible loss rate was 75.93 mL/day per L/min of sweep gas rate, regardless of the oxygenator size or patient weight [4]. Although there are subtle differences in reported loss rates, most studies reveal an insensible water loss arising from the ECMO oxygenator of roughly 3 mL/h per 1 L/min of sweep gas rate or 72 mL/h per 1 L/min, regardless of oxygenator size. Therefore, this estimate of insensible water loss could be utilized to estimate fluid losses for patients undergoing ECMO and thereby provide a more accurate assessment of a patient’s overall fluid balance. Despite the utility of the above analysis, it is still unclear whether this more comprehensive approach will ultimately result in better patient outcomes. It would be hard to envision a scenario where that would not be the case, but further studies that elucidate the clinical impact of accounting for insensible water loss are necessary before definitive conclusions can be drawn.

## Data Availability

The research data are available on request from the authors.
